# Research on the Reusability of Bentonite Waste Materials for Residual Chlorine Removal

**DOI:** 10.3390/ma17225647

**Published:** 2024-11-19

**Authors:** Ieva Andriulaityte, Marina Valentukeviciene, Ramune Zurauskiene

**Affiliations:** 1 Department of Environmental Protection and Water Engineering, Faculty of Environment Engineering, Vilnius Gediminas Technical University, LT-10233 Vilnius, Lithuania; ieva.andriulaityte@vilniustech.lt; 2 Department of Landscape Management and Agribusiness Technologies, Faculty of Agrotechnologies, Vilniaus Kolegija, Higher Education Institution, Dvaro g. 1, LT-14165 Vilnius, Lithuania; ramune.zurauskiene@vilniustech.lt; 3 Department of Building Materials and Fire Safety, Faculty of Civil Engineering, Vilnius Gediminas Technical University, LT-10233 Vilnius, Lithuania

**Keywords:** recycled materials, stormwater, residual chlorine, green infrastructure

## Abstract

Recyclable construction waste can be used as a low-cost material to reduce stormwater pollution caused by various pollutants. In recent years, studies have reported increased water contamination from chlorine and chlorine compounds and its negative impact on aquatic ecosystems. When assessing the need for waste recycling, circularity, and stormwater reuse, it is worth evaluating the capacities of construction waste materials to reduce stormwater pollution from residual chlorine. Laboratory experiments using bentonite waste material (bentonite clay) and sodium hypochlorite solutions were carried out to analyze the potential of bentonite clay to retain residual chlorine in stormwater and evaluate its capacity to be applied as filtration media in green infrastructure. In the first stage, the particle size distribution and texture of bentonite clay were assessed using laboratory sieve analysis and microscopy. The results of the experiments indicated that the optimal grain size to retain pollutants was 0.8–2.0 mm. The microstructure analysis showed the capacity of bentonite to retain residual chlorine. The results of the static and dynamic experiments (leaching and filtration tests) show that the bentonite clay retained up to 44% of the residual chlorine. The obtained results indicate that bentonite clay might be suitable for application as filtration media in green infrastructure to reduce stormwater contamination.

## 1. Introduction

Construction and demolition waste accounts for more than a third of all the waste generated in the EU [1]. Following the principles of circularity, action is required to reduce the amount of waste through increasing the levels of reuse and recycling [2]. Construction and demolition waste should be managed in a sustainable manner, assessing their added environmental and economic benefits [3]. For instance, they can be used as low-cost and recyclable filtration materials in stormwater treatments to retain harmful pollutants.

Stormwater contamination remains an environmental challenge that requires additional efforts and sustainable treatment solutions. Studies have shown that the increasing pollution from untreated stormwater contaminants has a negative impact on aquatic ecosystems and deteriorates the quality of surface water [4,5,6]. Since the beginning of the COVID-19 pandemic, environmental pollution from chlorine and chlorine compounds has grown [7,8]. Intensive outdoor space disinfection using chlorine-based disinfectants (e.g., sodium hypochlorite; NaOCl) has increased the concentrations of residual chlorine and other chlorine compounds in rivers and lakes [9,10,11]. Studies have reported that even small amounts of chlorine pose a risk to water ecosystems, decreasing the water quality, affecting aquatic flora and fauna [12], and affecting the corrosion level of outdoor coatings and structures [13,14]. In this regard, water contamination with chlorine and chlorine compounds is becoming an increasingly significant environmental issue [15,16,17]. The negative and harmful impacts of NaOCl on the environment, especially aquatic ecosystems, has attracted researchers’ interest and has increased the need to research and evaluate possible methods to reduce residual chlorine concentrations in stormwater. Previous experiments have reported that residual chlorine remains active for 14 days, depending on its concentration [12]. In view of this, residual chlorine should be retained at its source to prevent it from being carried into the environment. The European Commission directives report that even small amounts of micro-pollutants may pose a risk [18]. Green infrastructure can be an efficient barrier to minimize pollution in stormwater. Studies recommend including low-cost, waste, and recyclable materials in green infrastructure [19,20]. This would contribute to sustainability, circularity, and it adheres to the Green Deal policy, while reducing environmental pollution.

Studies focusing on water demand, water scarcity, and water quality issues have addressed solutions such as stormwater life cycle assessment and stormwater recycling [21,22]. This raises the need to apply the principles of the circular economy and sustainability in stormwater treatment areas contaminated by chlorine and chlorine compounds. Some solutions may be to focus on the use of construction waste materials for the retention of residual chlorine and on the recycling of polluted stormwater. From a global perspective, there is growing interest in meeting environmental challenges through green infrastructure (GI) and mitigating stormwater pollution at its source [23]. Studies on LCAs have presented GI as an effective solution for reducing stormwater pollution [5,24]. GI is defined as “a strategically planned network of natural and semi-natural areas with other environmental features designed and managed to deliver a wide range of ecosystems services” [25]. It is applied as an alternative option to recycle stormwater contaminated by harmful substances. GI includes technologies such as green roofs, wetlands, wet walls, rain gardens, and so on, and is applied to nature-based solutions aimed at treating stormwater and retaining pollutants before they are released into local water bodies [26]. The efficiency of GI in reducing stormwater pollution depends on the technical parameters of the construction layers (filtration, drainage, and plants). Filtration materials work in reactive barriers to mitigate pollutant and treat stormwater. The purpose of the drainage layer is to improve root breathing, eliminate weeds, and provide porosity and rot resistance. Filtration and drainage materials should have the following characteristics: granulometric composition, frost resistance, water permeability, structure and layer stability, reactive to compressive loads, and a certain pH value and salt content [27]. The efficiencies of different low-cost filter media to remove pollutants from stormwater have been widely analyzed in recent years [28,29].

In recent years, the increased use of bentonite clay in the construction industry has generated a huge amount of waste that, according to European legislation, should be reused and recycled [2]. In connection with this, studies have aimed to analyze the efficiency of bentonite clay in retaining residual chlorine and reducing stormwater pollution. Bentonite clay is a natural, environmentally friendly, and recyclable product that is widely used in the construction industry. Considering its physical and chemical features, it can be used to produce layers for drainage systems, insulation, building construction, sidewalks, bricks, and impermeable layers for landfills, among other uses [30,31]. Bentonite clay contains about 60–70% montmorillonite mineral, and has high absorption, catalytic, and binding capacities [32]. These features make bentonite an efficient material to improve soil and water quality by retaining harmful pollutants [33,34].

The novelty of this research is that it investigated the possibility of using recyclable construction waste material (bentonite clay) for stormwater treatment, evaluated its efficiency in retaining residual chlorine, and provides recommendations for applying the tested construction material in green infrastructure as a filtration layer to reduce stormwater pollution from residual chlorine.

## 2. Materials and Methods

**Figure 1:**
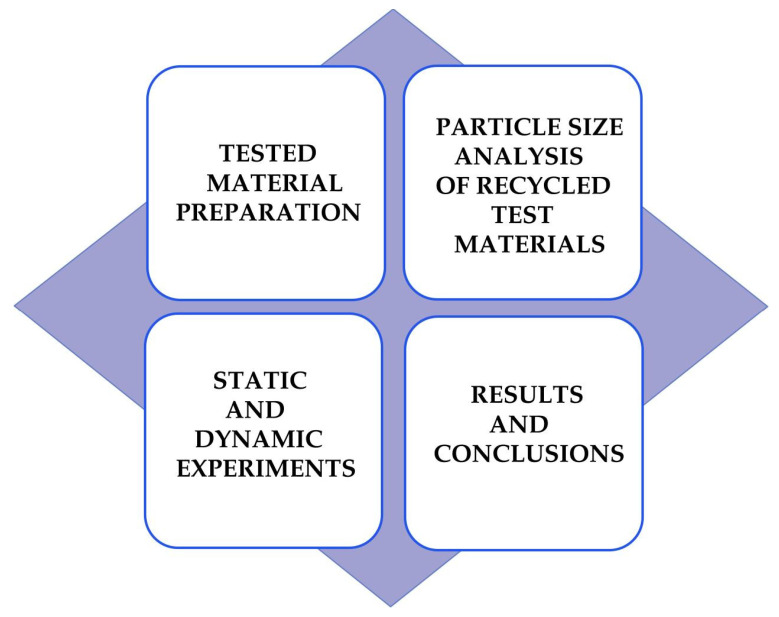
Order of experiments.

The experiments were carried out in the following order (Figure 1): the test materials were prepared (bentonite clay, stormwater); particle size analysis (sieve test, microscopy) was carried out on the test material (bentonite clay) to assess its suitability for the removal of pollutants; and static and dynamic experiments (leaching and column tests) were carried out to evaluate the efficiency of bentonite clay to retain residual chlorine and reduce stormwater pollution.

The following materials and equipment were used in the experiments:

- Bentonite clay samples (fractions of 0.8–2.0 mm) (waste material from building construction);
- Sodium hypochlorite solution (1000 ppm);
- Stormwater samples (collected from disinfected areas);
- Filtration column, mixer, and glass jars (6 units);
- Sieves (7 units);
- Microscope (Celestron, Digital Microscope Imager, Biolight 200, Celeston, Torrance, CA, USA);
- Laboratory scales (Kern PCB, max of 2500 g d = 0.01 g; KERN PCB-FACTORY GmbH, Regensburg, Germany);
- Spectrophotometer (Thermo Scientific Genesys 10 S, Waltham, MA, USA);
- A portable conductivity measurement device (Cond315i, WTW, Weilheim, Germany) and pH-meter instrument (WTW pH 323, WTW, Weilheim, Germany);
- A chlorine meter (CL200 ExStik, measuring range of 0.01 ppm–10 ppm, accuracy of ±10% for reading ±0.01 ppm, temperature range of −5 to +90 °C, automatic self-calibration; Extech, Beijing, China).

### 2.1. Sieve Test and Microscopy

The experiments were carried out using waste bentonite clay. The particle size of the bentonite clay was evaluated using a sieve analysis test and microscopy. Bentonite clay belongs to the third group of clays, which are characterized by their high water absorption and swelling capacities. Due to these properties, bentonite clay is widely used in the construction sector, mainly for drilling and sealing, as well as a binder or adsorbent. Bentonite clay is an effective material for removing pollutants and is applied in stormwater treatments to retain contaminants and reduce water turbidity [35]. The experiments were carried out using dry granules of bentonite clay. Bentonite contains 40–50% SiO_2_, 20–30% Al_2_O_3_, 3–6% Fe_2_O_3_, and small amounts of CaO, Na_2_O, MgO, and K_2_O.

**Figure 2:**
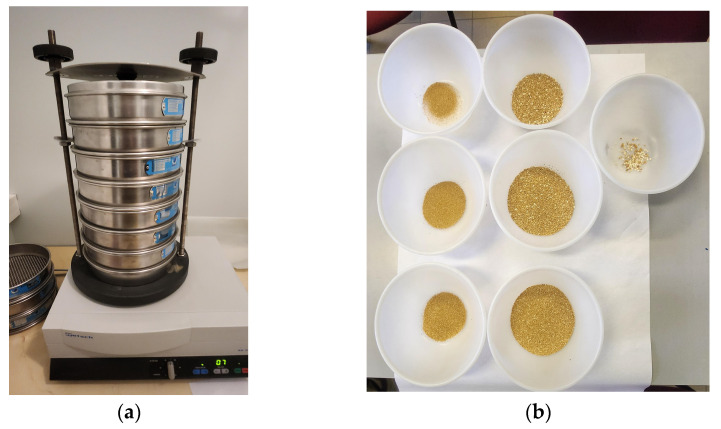
Sieve analysis test: (**a**) seven calibrated sieves with mesh sizes of 500 µm, 600 µm, 800 µm, 1000 µm, 1600 µm, 2000 µm, and 2500 µm; (**b**) testing material after separation through the sieves.

A study showed that laboratory sieve analysis is the most commonly applied method to measure the particle size distribution of tested samples [36]. A granulometric analysis of bentonite clay was carried out to verify its suitability for application in green infrastructure as a filtration layer to retain residual chlorine. Seven calibrated sieves with mesh sizes of 500 µm, 600 µm, 800 µm, 1000 µm, 1600 µm, 2000 µm, and 2500 µm and laboratory scales (Kern PCB, max of 2500 g d = 0.01 g) were used for the analysis (Figure 2).

The evaluation of the tested samples’ granulometric composition was performed as follows: 1000 g of dry material was weighed using a laboratory scale. Then, the weighed mass was poured into the top sieve and dispersed through the mesh’s opening. This process continued until a fixed mass was obtained on each sieve. Later, the remaining mass was weighed, and the percentage was calculated. The test materials are described using the following parameters: minimum grain diameter—d_min_ (mm); maximum grain diameter—d_max_ (mm); non-uniformity coefficient of the filler—K_60_; effective diameter of filtered media grains—d_v_ (mm); filter media height—h (mm). The K_60_ coefficient was calculated using the following formula:

K_60_ = d_60_/d_10_ (1)

where d_60_ is the diameter of the sieve mesh holes through which 60% of the granular material mass of the filter media was sifted, and d_10_ is the diameter of the sieve mesh holes through which 10% of the granular material mass of the filter material was sifted. The effective diameter of the filter media grains—d_v_ (mm)—was calculated using the following formula:

d_v_ = (d_10_ + d_60_)/2 (2)

The obtained results were used to draw a particle size distribution curve for the tested material grains. After the sieves were analyzed, four samples were selected (0.8–2.0 mm).

Microscopy was carried out to evaluate the surface particle structure of the bentonite clay (enlarged 80 times). The bentonite clay test samples were analyzed before and after the filtration of stormwater polluted with sodium hypochlorite. The analysis aimed to determine the changes in the surface pore structure of the bentonite clay before and after its interaction with chlorine. Visual evaluation using microscopy is important for understanding the capacity of bentonite to retain chlorine and define the changes incurred after its interaction with a sodium hypochlorite solution.

### 2.2. Static and Dynamic Experiments

Static and dynamic experiments (leaching and filtrations) were carried out to evaluate the efficiency of low-cost, recyclable construction material (bentonite clay) in retaining residual chlorine in stormwater, and thus, its suitability for application in green infrastructure as a filtration layer. Raw stormwater test samples were prepared at the laboratory and the following indicators were measured: pH, conductivity (µS/cm), turbidity (NTU), and color (AV). Then, the stormwater samples were mixed with a sodium hypochlorite solution, based on WHO recommendations (1000 ppm), and their pH, conductivity (µS/cm), turbidity (NTU), color (AV), and residual chlorine concentration (ppm) were measured. Different concentrations of a disinfectant mixture were used to evaluate quality changes in the stormwater.

A leaching test was carried out to analyze the release of residual chlorine into the environment from outdoor surface coatings and to evaluate the efficiency of bentonite clay in retaining residual chlorine. Six glass jars with a size of 1000 mL were used for the experiment: one glass jar was filled with raw stormwater (control sample), and the other five were filled with a 2 cm layer of bentonite clay and stormwater samples polluted with sodium hypochlorite. Then, all six samples underwent mixing at a speed of 120 rpm for 30 min, according to a previous protocol.

After mixing for 30 min, the pH, conductivity (µS/cm), turbidity (NTU), color (AV), and concentration of residual chlorine (ppm) of the water samples were measured. The experiment was repeated three times.

**Figure 3:**
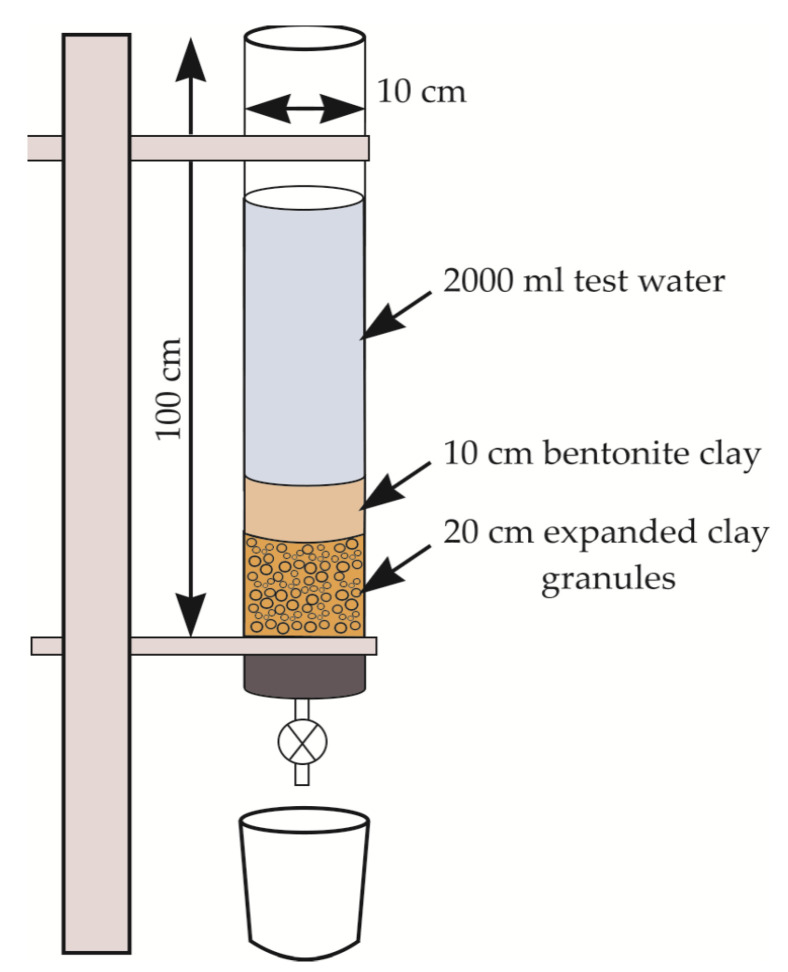
Scheme of filtration test set-up.

A filtration test was performed using a glass column (height of 100 cm, diameter of 10 cm), recyclable construction waste material (bentonite clay in fractions of 0.8–2.0 mm), expanded clay aggregate, and test water samples (raw stormwater sample and test samples polluted with sodium hypochlorite) (Figure 3). The experiment was carried as follows: In order to determine the water quality indicators of the test samples, a control test was performed. A glass column was filled with 20 cm of expanded clay aggregates as a supporting layer and 10 cm of the recyclable material (bentonite clay) as filter media, topped with 2000 mL of the raw test water samples, and left for 30 min. After 30 min, the test water was collected in a reservoir, and the quality indicators (pH, conductivity, turbidity, and color) were measured. The experiment was repeated three times to obtain reliable results.

In the next stage, test water samples were polluted using different concentrations of sodium hypochlorite and added to a glass column filled with 20 cm of expanded clay granules (supporting layer) and 10 cm of bentonite clay. Different concentrations (250 ppm, 500 ppm, 750 ppm, and 1000 ppm) were used to evaluate how disinfection impacted the stormwater quality indicators (pH, conductivity, turbidity, and color). After 30 min of contact time, the pH, conductivity, turbidity, and color of the collected filtered water samples were measured. The experiment was repeated three times.

## 3. Results and Discussions

### 3.1. Sieve Analysis and Microscopy

**Figure 4:**
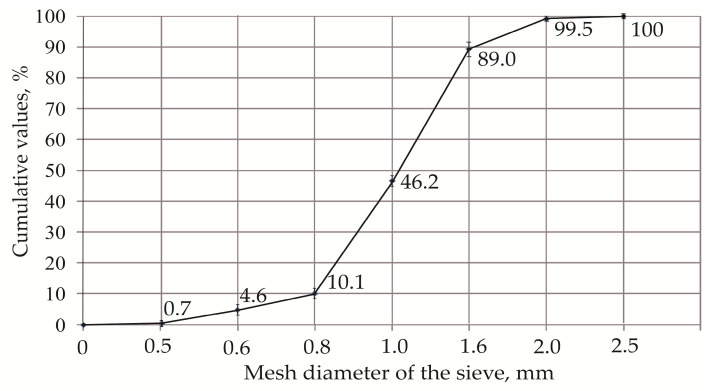
The distribution curve of the bentonite clay’s grain sizes.

The aim of this research was to evaluate the possibility of using waste and reusable bentonite clay in green infrastructure as a filtration layer (between the substrate and drainage layers). The main function of the filtration layer is to retain pollutants (residual chlorine) and to prevent the soil fines from entering and contaminating the drainage layer. Granulometric analysis (sieve analysis) can be used to evaluate the grain size of bentonite clay to prevent small particles from polluting and clogging the drainage layer in green infrastructure. Studies have shown that the grain size is a significant characteristic in terms of a particle’s capacity to retain contaminants. The efficiency of a pollutant removal process depends on the particle sizes and on the interaction between the particles and the filtering substance. Smaller grains have a collectively larger surface area and a higher capacity to retain contaminants. Undersized particles can cause problems such as filter blockage and changes in the flow rate and the filtration capacity. Higher porosities have been demonstrated to have better contaminant retention capacities [37]. The obtained results of the sieve analysis are graphically represented as a particle size distribution curve in Figure 4.

**Figure 5:**
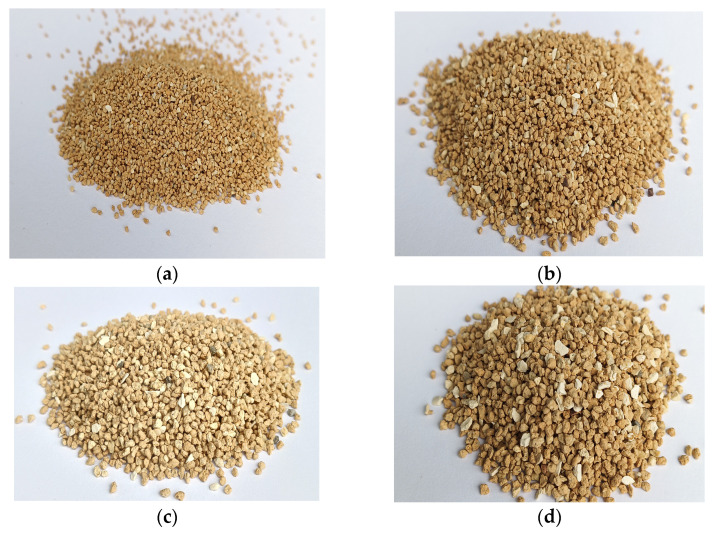
Test samples of bentonite clay suitable for filtration: (**a**) 0.8 mm; (**b**) 1.0 mm; (**c**) 1.6 mm; (**d**) 2.0 mm.

Figure 4 presents the bentonite clay‘s grain size distribution in relation to its mass. The distribution curve shows that fractions of bentonite clay could be applied as a filtration layer. The graph shows that only 0.7% of the bentonite material passed through the sieve with a mesh diameter of 0.5 mm. This indicates that only insignificant fractions were smaller than the sieve size used. The sieve with a mesh diameter of 2.5 mm showed that 100 percent of the bentonite particles passed through the sieve, indicating the largest particle size in the tested sample. The distribution curve indicates that the optimal size of bentonite grains for the removal of pollutants is 0.8–2.0 mm (Figure 4). Figure 5 presents the particle sizes of the selected samples.

**Figure 6:**
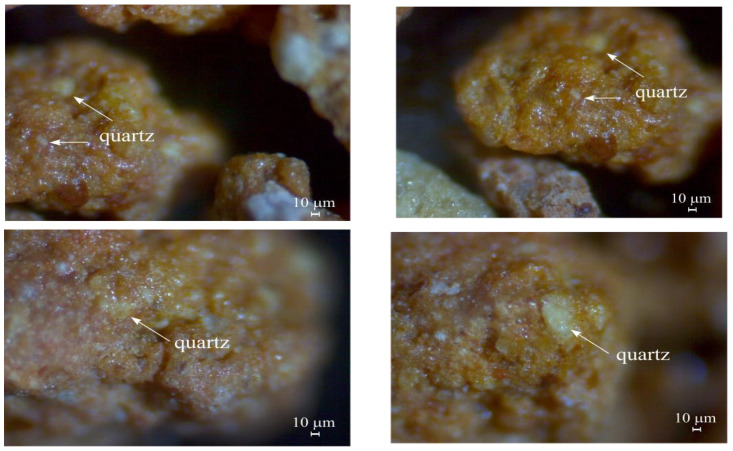
Microstructure analysis of the surface structure of bentonite clay before interacting with the sodium hypochlorite solution (enlarged 80 times).

**Figure 7:**
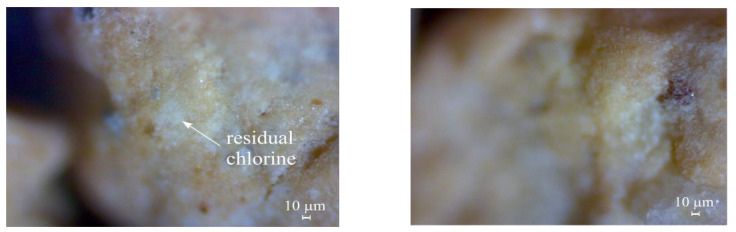

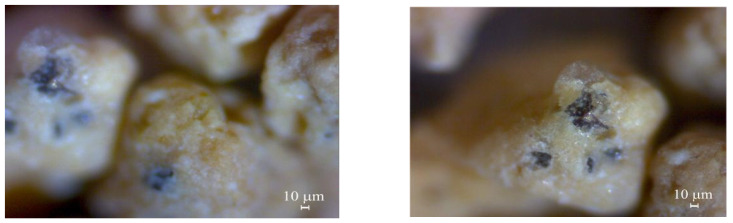
Microstructure analysis of bentonite clay surface structure after interacting with sodium hypochlorite solution (enlarged 80 times).

Microstructure analysis of the particle structure of the test material (bentonite clay) was carried out using microscopy. Changes in the bentonite clay surface before and after its interaction with the sodium hypochlorite solution were observed. Figure 6 (before filtration) and Figure 7 (after filtration) present the microscopy results (enlarged 80 times).

Figure 6 shows the surface of the bentonite clay particles, where clearly visible sintered clay compounds have bound together with quartz particles. The particle surface of the tested material was uneven, covered with small depressions and pores, and had a large surface area. This creates favorable conditions for the attachment of particles passing by and the formation of a new surface layer. These characteristics may lead to the effective retention of pollutants in stormwater.

The changes in the bentonite surfaces that occurred after filtration of the stormwater sample polluted with disinfectant (sodium hypochlorite, 1000 ppm) are presented in Figure 7.

There were visible changes in the bentonite granule surface structure after interacting with the sodium hypochlorite solution. Figure 7 shows that the entire surface of the bentonite clay was covered in a white sediment layer with a thickness of up to 1 mm. It can be concluded that the formation of the sediment layer was caused by the uneven, rough, and large surface area of the material. These results reveal the capacity of bentonite clay to retain residual chlorine.

### 3.2. Static and Dynamic Experiments

We continued an experiment from previous research focused on the evaluation of the capacities of low-cost, local, and recyclable materials to remove residual chlorine from stormwater [20]. According to the goals of sustainability and circularity and recommendations for construction waste recycling [2,38,39], the experiments were carried out to evaluate the efficiency of bentonite clay in retaining residual chlorine, in order to recommend its reuse and application in green infrastructure as a filtration layer placed under soil substrate. Hence, leaching and filtration tests were conducted.

Leaching test. The efficiency of bentonite clay in retaining residual chlorine was tested by mixing it with stormwater samples at 120 rpm for 30 min. Six glass jars with a volume of 1000 mL were used: one glass jar was used as a control sample (the initial stormwater sample mixed with sodium hypochlorite at a concentration of 1000 ppm), and the other five glass jars were filled with a 2 cm layer of bentonite clay and stormwater samples polluted with sodium hypochlorite (concentration of 1000 ppm). After 30 min of mixing, the following stormwater indicators were measured in the tested samples: pH, conductivity (μs/cm), turbidity (NTU), color (AV), and the concentration of residual chlorine (ppm). In order to compare the changes in the stormwater indicators (pH, conductivity, turbidity, and color) after the test water samples were in contact with the bentonite clay, a control test was conducted (the initial stormwater test sample). The following values were obtained: pH—7.5; conductivity—2.3 μs/cm; turbidity—0.051 NTU; and color—1.029 AV. The stormwater indicators of the test water samples with 1000 ppm of sodium hypochlorite were as follows: pH—8.06; conductivity—49.4 μs/cm; turbidity—0.006 NTU; color—0.069 AV (Table 1). These results can be explained by the release of residual chlorine in the test water sample. These slight changes can impact water quality and aquatic ecosystems. Significant changes in the conductivity (about 20 times higher values) were reported for the stormwater polluted with sodium hypochlorite. The conductivity of the water sample increased from 450 µS/cm to 600 µS/cm, indicating a rise in the concentration of dissolved ions. The results of the leaching test are presented in Table 1.

Table 1 shows that the pH values of the polluted samples varied between 9.79 and 10.49 and increased by about 1.2–1.3 times compared to the test sample with sodium hypochlorite before contact with bentonite clay and by about 1.3–1.5 times compared to the values of the initial stormwater indicators. Meanwhile, the increases in the conductivity after the test water samples were in contact with the bentonite were significant at about 1.97–5.7 times (97.2–285.0 µS/cm) higher values compared to the sample before contact and the initial stormwater. The turbidity values after mixing showed slight increases of about 0.041–0.611 NTU compared to the samples before contact (0.069 NTU) and the initial stormwater sample (0.051 NTU). The color value changed by about 1.3 times compared to the test sample before mixing. These changes in the stormwater indicators may be due to the contact of the tested solution with bentonite clay and the possible resulting reactions. After mixing for 30 min, the bentonite clay showed a residual chlorine retention efficiency of up to 44%. These results show that bentonite clay could be applied as a filtration layer in green infrastructure. The different retention values obtained depended on the bentonite grains size and on possible accumulation effects.

During later experiments, the bentonite clay used for the leaching test was washed with distilled water, and the water indicators and residual chlorine were measured to determine how much residual chlorine remained on the surface. The results revealed residual chlorine concentrations of 0.13–0.26 ppm. The stormwater indicator values were detected as follows: pH value—9.727–9.351 (control value of 7.99); conductivity value—55.5–62.9 µS/cm (control value of 3.6 μs/cm); turbidity value—0.233–0.294 NTU (control value of 0.060 NT); and color value—1.223–1.230 AV (control value of 1.040 AV). These results indicate that residual chlorine remains on outdoor coatings for some time and poses a negative risk to the environment [17].

**Figure 8:**
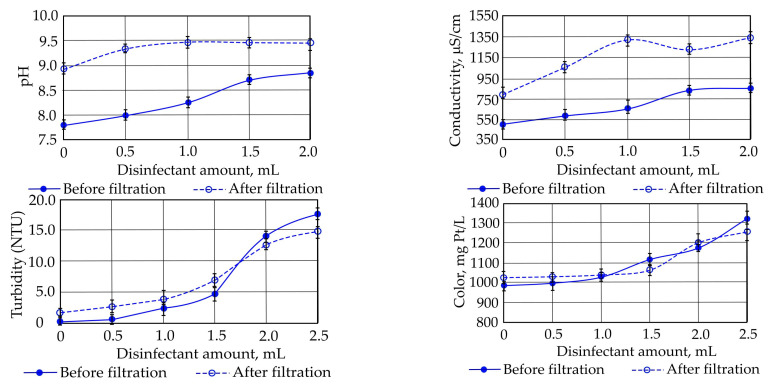
Changes in stormwater indicators depending on disinfectant concentrations.

Filtration test. Samples of the initial stormwater were used for a control test to measure the main stormwater indicators (pH, conductivity, turbidity, and color). The obtained results were as follows: pH—8.12; conductivity—2.2 µS/cm; turbidity—0.061 NTU; and color—1.042 AV. A filtration test was conducted using 20 cm of expanded clay aggregate as the supporting layer (with a fraction size of 8.0–11.0 mm) and 10 cm of bentonite clay as the filtration layer (with a fraction size of 0.8–2.0 mm). The solution was prepared by mixing the stormwater samples with different concentrations of sodium hypochlorite (250 ppm, 500 ppm, 750 ppm, and 1000 ppm). Different concentrations of sodium hypochlorite were used to evaluate how disinfection impacts the stormwater indicators. The control test showed that the filtration of the stormwater samples using bentonite clay changed the values of the stormwater indicators. The obtained pH value of the initial stormwater sample was 8.12 before filtration and 9.77 after filtration. The conductivity of the test sample was detected as 2.2 µS/cm before filtration and 129.3 µS/cm after filtration. The low value of the initial sample (2.2 µS/cm) indicates that the water was not contaminated or only slightly contaminated. The significant increase in the conductivity after filtration could be because, during the filtration process, the bentonite clay released some substances that dissolve in water, raising its conductivity. The turbidity and color values changed slightly: the turbidity increased from 1.04 to 1.14 NTU, and the color changed from 0.06 to 0.14 AV. It can be concluded that some bentonite particles were washed into the water and caused the changes in the turbidity and color. Figure 8 presents the changes in the stormwater indicators depending on the amount of disinfectant. The pH value increased after filtration to about 9.0 and remained stable even with increases in the amount of sodium hypochlorite. It can be concluded that the sodium hypochlorite affected the water chemical composition, which may be related to the reactions between the disinfectant and water components. After filtration, the pH was higher and more stable because filtration can remove or neutralize certain substances, leaving a more alkaline water residue. This indicates that the filtration process effectively stabilized the pH even with higher amounts of disinfectant. The conductivity before and after filtration changed slightly. The increases depended on the disinfectant’s concentration, and it may have also been affected by the contact of the solutions with the bentonite clay.

The changes in the turbidity indicate that after the contact of the sodium hypochlorite solution with bentonite clay, the residual chlorine reacted with the bentonite clay, resulting in the formation of precipitates. Higher amounts of disinfectant led to increases in the color value. This may be because the disinfectants in contact with bentonite clay formed colored compounds. Chlorine reacts with bentonite and oxidizes surface substances, changing the color of the test water samples. The analysis results of the filtration processes of the test water samples with a concentration of 1000 ppm indicated that the pH value was 9.21 before filtration and 9.83–10.92 after filtration. This is because the filtration through bentonite clay caused an increase in the alkalinity of the tested samples (Table 2).

Table 2 shows that the initial water conductivity detected was 2.2 µS/cm before filtration and 129.3 µS/cm after filtration. The low value of the initial sample (2.2 µs/cm) indicates that the water was not contaminated or only slightly contaminated. The significant increase in conductivity after filtration might be because, during the filtration process, the bentonite clay released some substances that dissolved in the water and raised the conductivity. The solution’s conductivity before filtration was 49.4 µS/cm. Compared to the value of the initial sample (2.2 µS/cm), this indicates contamination of the water with sodium hypochlorite. After filtration, the conductivity’s value was found to have decreased from 172.3 to 130.8 µS/cm. It can be concluded that the filter material retained contaminants and dissolved substances. Table 2 shows that there were significant changes in the turbidity and color values. The values of the residual chlorine after filtration indicate a chlorine retention efficiency of bentonite clay of up to 44%. Previous research presented differences in the ability of lightweight aggregates to remove residual chlorine from stormwater compared to the obtained capacity of bentonite clay [20].

## 4. Conclusions

1. Following the principles of circularity, recycled construction waste could be used as a low-cost material to reduce stormwater polluted with residual chlorine. The application of construction waste materials in green infrastructure as a filter layer is an innovative and sustainable solution to reduce stormwater contamination.
2. The sieve analysis indicated that the optimal size of bentonite clay to retain pollutants is 0.8–2.0 mm.
3. The microscopy analysis of bentonite clay before and after its interaction with sodium hypochlorite solution showed visible changes in the bentonite surface structure. A white color and a layer up to 1 mm thick appeared on the bentonite surfaces after the filtration process, indicating the capacity of bentonite clay to retain residual chlorine.
4. The static and dynamic experiments (leaching and filtration tests) reported a residual chlorine retention efficiency of bentonite clay of up to 44%. The experiments revealed that bentonite clay might be suitable for application as one of the filtration layers in green infrastructure to reduce stormwater pollution from residual chlorine.
5. It is recommended to continue investigations with other construction waste materials suitable for Green Deal recommendations, in order to decrease the amount of waste from construction and to encourage recycling processes.

## Figures and Tables

**Table 1:** Leaching test.

Indicator	I	II	III	IV	V	VI
pH	8.06	10.39	10.23	10.49	9.79	9.99
Conductivity, µS/cm	49.4	285.0	161.0	265.0	105.1	97.2
Color, AV	1.052	1.412	1.358	1.611	1.239	1.254
Turbidity, NTU	0.069	0.041	0.368	0.611	0.245	0.265
Residual chlorine,	3.14	2.04	1.76	1.75	2.51	2.63
ppm						

Notes: I—stormwater mixed with sodium hypochlorite before contact with bentonite clay; II, III, IV, V, VI—stormwater mixed with sodium hypochlorite after contact with bentonite clay.

**Table 2:** Filtration test.

Indicator	I	II	III	IV	V	VI	VII	VIII
pH	8.12	9.77	9.21	9.86	9.83	10.10	10.18	10.32
Conductivity, µS/cm	2.20	129.3	49.4	172.3	143.9	138.2	136.4	130.8
Color, AV	0.06	0.14	0.10	0.11	0.11	0.11	0.11	0.11
Turbidity, NTU	1.04	1.14	1.05	1.10	1.10	1.10	1.10	1.10
Residual chlorine,	-	-	3.21	2.92	1.96	1.92	1.79	1.91
ppm								

Note: I—initial sample; II—initial sample after filtration; III—sample mixed with sodium hypochlorite before filtration; IV, V, VI, VII, VIII—samples mixed with sodium hypochlorite after filtration.

## Data Availability

The original contributions presented in the study are included in the article; further inquiries can be directed to the corresponding author.
